# Supplementary figures and images for: Tissue resident memory T cells populate the human uveal tract
Source: Sci Rep. 2026 Apr 3;16:11330. doi: 10.1038/s41598-025-33444-2 (PMC13049005; doi:10.1038/s41598-025-33444-2)

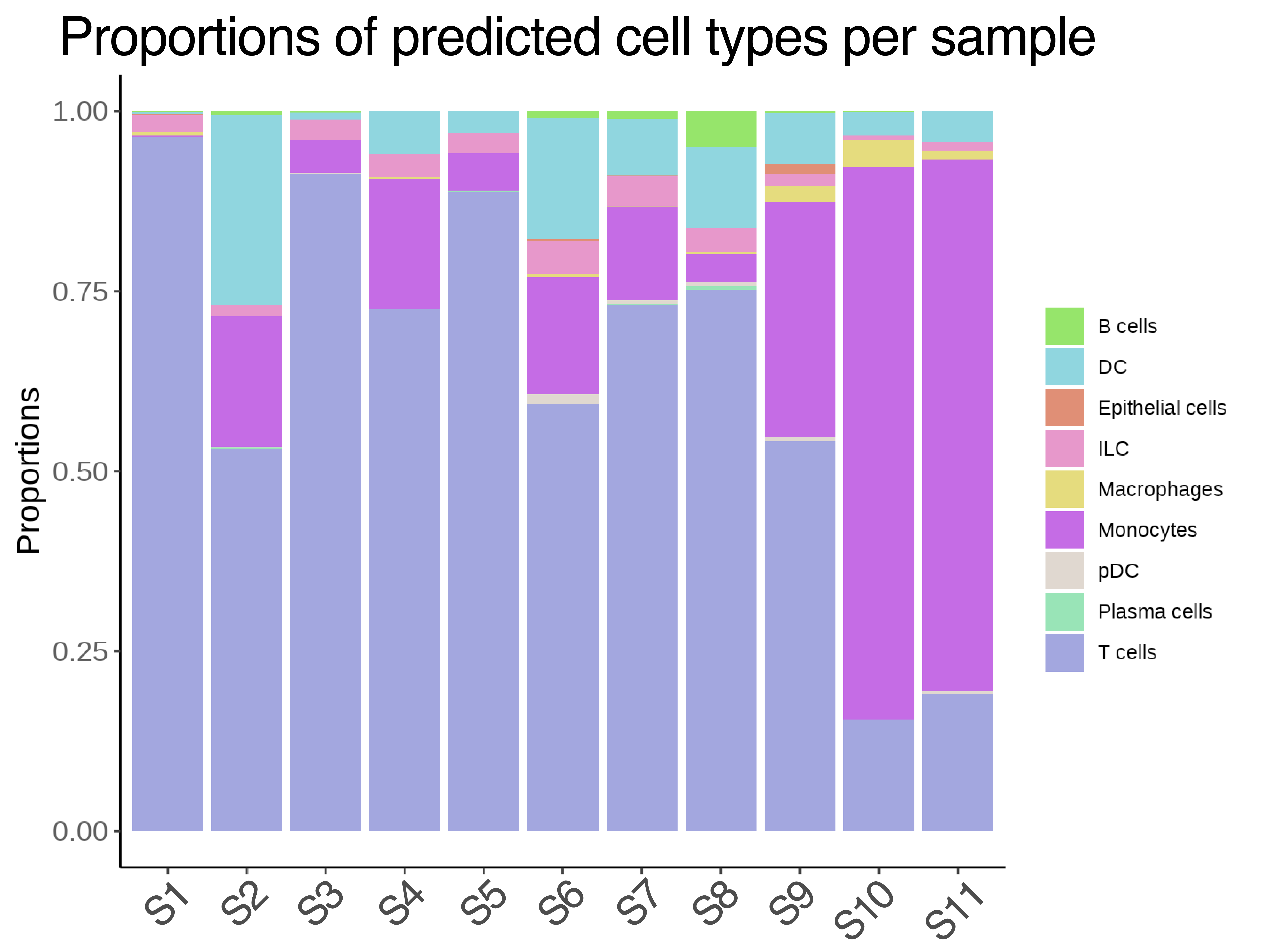

Supplement: Supplementary file 1 — Supplementary Material 1 [file 41598_2025_33444_MOESM1_ESM.zip › Supplementary_figure_2.png]

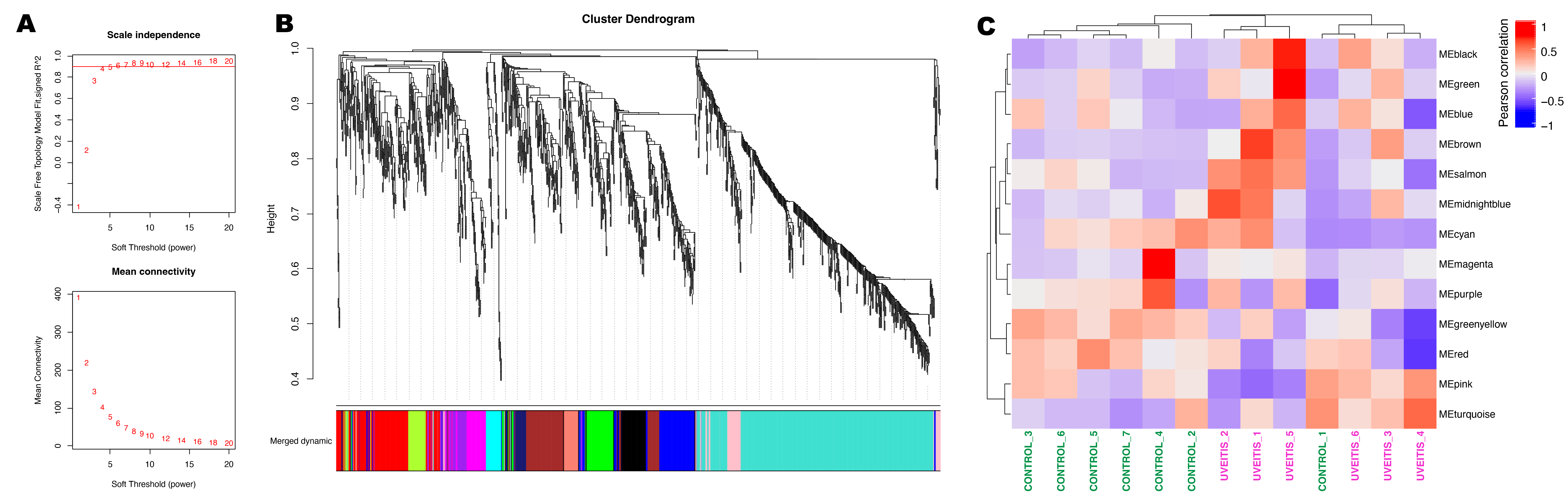

Supplement: Supplementary file 1 — Supplementary Material 1 [file 41598_2025_33444_MOESM1_ESM.zip › Supplementary_figure_4.png]

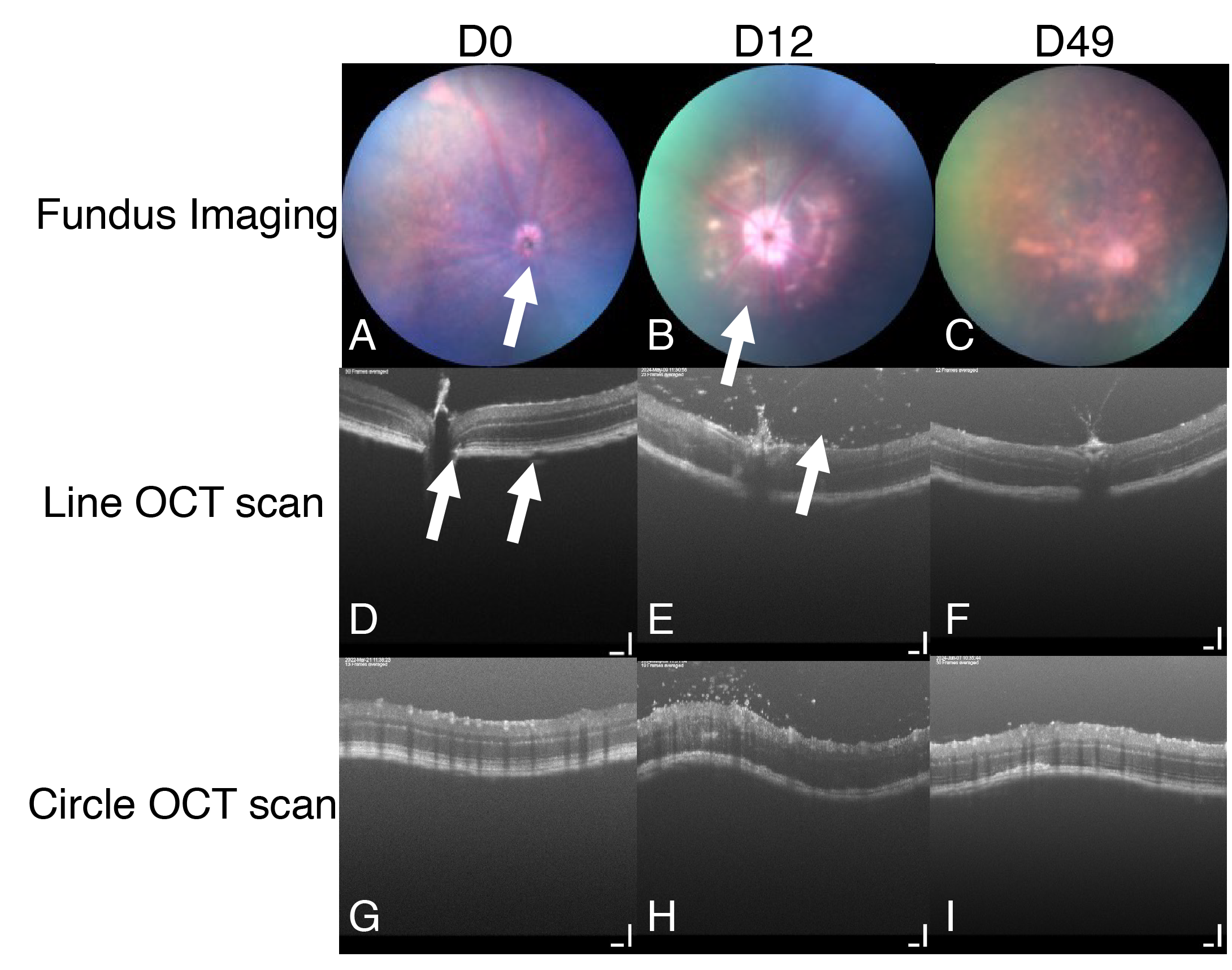

Supplement: Supplementary file 1 — Supplementary Material 1 [file 41598_2025_33444_MOESM1_ESM.zip › Supplementary_figure_5.png]
